# From pathophysiology to therapy: dual and triple agonists as a new frontier in metabolic dysfunction-associated steatotic liver disease

**DOI:** 10.3389/fphar.2026.1784959

**Published:** 2026-10-13

**Authors:** Christiano Argano, Dalila Maggio, Chiara Pollicino, Virginia Cangialosi, Stefania Biscaglia Manno, Valentina Orlando, Martina Lucia Monti, Arianna Grutta, Alessandra Torres, Salvatore Corrao

**Affiliations:** 1 Department of Internal Medicine, National Relevance and High Specialization Hospital Trust (ARNAS Ospedali Civico Di Cristina Benfratelli), Palermo, Italy; 2 Department of Health Promotion Sciences, Maternal and Infant Care, Internal Medicine and Medical Specialties (PROMISE), University of Palermo, Palermo, Italy

**Keywords:** cotadutide, dual agonists, MASLD, pan-PPAR agonists, retatrutide, survodutide, tirzepatide, triple agonists

## Abstract

Metabolic dysfunction-associated steatotic liver disease (MASLD) has emerged as the most prevalent chronic liver condition worldwide, reflecting the growing global burden of obesity and type 2 diabetes (T2D). The complex pathophysiology of MASLD, characterized by insulin resistance (IR), lipotoxicity, and chronic low-grade inflammation, necessitates therapeutic strategies that address systemic metabolic dysfunction rather than isolated hepatic markers. Current evidence highlights the synergistic potential of targeting multiple incretin and metabolic pathways. Dual agonists, most notably Tirzepatide (a GIP and GLP-1 receptor agonist), have demonstrated profound efficacy in clinical trials (SURPASS and SURMOUNT programs), achieving significant reductions in hemoglobin A1c, body weight, and liver fat content. Furthermore, dual GLP-1/glucagon receptor agonists like Survodutide and Cotadutide offer complementary benefits by enhancing energy expenditure and modulating hepatic lipid metabolism. The development of triple agonists, such as Retatrutide (targeting GIP, GLP-1, and glucagon receptors), represents a further escalation in potency, showing unprecedented results in liver fat reduction and weight loss. The integration of these multi-target agents, together with emerging pathways involving amylin analogues and pan-PPAR agonists like Chiglitazar, marks a paradigm shift toward holistic cardiometabolic management. These therapies not only target the resolution of metabolic dysfunction-associated steatohepatitis and fibrosis but also address the interconnected risks of cardiovascular and renal complications. This review aims to explore the evolving landscape of pharmacological interventions, focusing on the emergence of dual and triple receptor agonists as a transformative frontier in metabolic dysfunction and MASLD therapy.

## 1 Introduction

Metabolic dysfunction associated steatotic liver disease (MASLD) has become extremely prevalent during the past decades and now represents the most common chronic liver disease worldwide. Currently, 38% of all adults and 7%–14% of children and adolescents have MASLD (Younossi et al., 2025) including approximately 60%–70% of individuals with T2D and approximately 70%–80% of those with obesity (Corrao et al., 2025). According to the updated Global Burden of Disease Study 2021 the global burden of MASLD increased from 1.69 million Disability-Adjusted Life Years (DALYs) in 1990 to 3.67 million DALYs in 2021 (i.e., approximately 2.2 times as many DALYs as there were 3 decades ago) (Tilg et al., 2025).

The Global Burden of Disease Study 2023 highlighted metabolic dysfunction-associated steatotic liver disease (MASLD) as one of the major emerging challenges in global public health. In 2023, approximately 1.3 billion people, corresponding to 16.1% of the world’s population, were estimated to be affected by MASLD, representing a 142.7% increase in the absolute number of cases compared with 1990. Although the age-standardized prevalence rate increased by 28.6% over the same period, the age-standardized burden measured in disability-adjusted life years (DALYs) remained relatively stable, suggesting improvements in the management of disease-related complications. Hyperglycaemia, high body mass index, and cigarette smoking were identified as the main modifiable risk factors contributing to the burden of MASLD. Projections indicate a further increase to approximately 1.8 billion cases by 2050 (+42% compared with 2023), largely driven by population growth and, to a lesser extent, by population ageing and epidemiological changes. These findings underscore the need for preventive strategies targeting cardiometabolic risk factors and for strengthening healthcare systems, particularly in regions with limited access to high-quality care (Kim et al., 2026).

By 2040, the MASLD prevalence rate for adults is projected to increase to more than 55% (Adinolfi et al., 2026) with higher prevalence among obese and diabetic individuals. In this sense, the epidemiological progression of liver steatosis is closely linked to the steady increase in diabetes and obesity (Roth et al., 2020; Zhou et al., 2024), determining an outstanding social, healthcare burden and hospitalization (Roth et al., 2020; Zhou et al., 2024; Bixby et al., 2019; Butt et al., 2024; Younossi et al., 2023a; Younossi et al., 2023b; Argano et al., 2022). The various forms of MASLD are associated with an elevated risk of progression to advanced fibrosis, cirrhosis, and hepatocellular carcinoma (Sheka et al., 2020; Powell et al., 2021). Individuals with Metabolic Dysfunction-Associated Steatohepatitis (MASH) and hepatic fibrosis are predisposed to an augmented risk of developing T2D, cardiovascular disease, chronic kidney disease, and different types of extrahepatic cancers (Stefan and Cusi, 2022; Targher et al., 2021; Targher et al., 2024). Furthermore, an escalation in fibrosis severity has been shown to be concomitant with an augmented risk of major adverse cardiovascular events (MACEs), thus underscoring the interconnectedness of clinical outcomes among metabolic syndrome and associated diseases (Simon et al., 2022).

MASLD, obesity, and T2D share a common pathogenesis having a fundamental connection in insulin resistance (IR) (Corrao et al., 2025; Chao et al., 2019; Licata et al., 2006). Physiologically, metabolic homeostasis is maintained by the liver, skeletal muscle, and adipose tissue, which sequester glucose and lipids within individualized thresholds. Insulin promotes glycogen synthesis and glucose uptake while inhibiting lipolysis in white adipose tissue, thereby limiting circulating free fatty acids (FFAs) (Chao et al., 2019; Santoro et al., 2021). In MASLD, exceeding adiposity thresholds triggers adipocyte IR (Lee et al., 2023). This impairs nutrient storage, causing an unregulated flux of FFAs and proinflammatory mediators to the liver (Corrao et al., 2025; Ciccarelli et al., 2023). Concurrently, hyperinsulinemia drives *de novo* hepatic lipogenesis, aggravating steatosis and fibrosis (Tamura and Shimomura, 2005). Steatosis further impairs the suppression of hepatic gluconeogenesis; combined with reduced muscle glucose disposal, this sustains hyperglycaemia and IR (Petersen et al., 2017). Consequently, agents targeting IR and the incretin system are under investigation for MASLD/MASH (Ciccarelli et al., 2023). These conditions are characterized by a blunted incretin effect (Petersen et al., 2017), potentially due to reduced endogenous Glucagon-Like Peptide-1 (GLP-1), impaired Gastric Inhibitory Polypeptide (GIP) signaling, and upregulated hepatic expression of dipeptidyl peptidase-4 (DPP-4) (Bernsmeier et al., 2014; Baumeier et al., 2017).

GLP-1 receptor agonists (GLP-1RAs) (Nauck et al., 2021), either alone or in combination with GIP (Corrao et al., 2024) and/or glucagon receptor agonists (Katsi et al., 2025), have now emerged as a promising form of pharmacotherapy for treating metabolic disorders, including MASLD/MASH, as well as obesity and T2D with beneficial effects on long-term adverse liver outcomes (Loomba et al., 2024; Sanyal et al., 2024a). A recent phase 2 trial involving participants with MASH and moderate or severe fibrosis showed that tirzepatide, for 52 weeks, was more effective than placebo with respect to resolution of MASH without worsening of fibrosis (Loomba et al., 2024). Another study showed the efficacy of Retatrutide, a novel triple agonist of the glucose-dependent insulinotropic polypeptide, glucagon-like peptide 1 and glucagon receptors, on liver fat reduction which was significantly related to changes in body weight, abdominal fat and metabolic measures associated with improved insulin sensitivity and lipid metabolism (Sanyal et al., 2024a).

The efficacy and safety of survodutide (a dual agonist of glucagon receptor and GLP-1 receptor) in people with MASH and liver fibrosis was evaluated in a phase 2 trial showing that it was superior to placebo with respect to improvement in MASH without worsening of fibrosis (Sanyal et al., 2024b). Moreover, experimental studies showed that PPARα/γ dual agonist determined a significant reduction in blood glucose and lipid levels (Laghezza et al., 2023). Given this background an extensive search of SCOPUS, PubMed, and CENTRAL was performed using the following string: (obesity OR diabetes OR MASLD OR Metabolic Dysfunction- Associated Steatotic liver Disease) AND (tirzepatide OR retatutride OR dual agonist OR triple agonist). The search string retrieved 3,090 manuscripts. Hand-searching of principal generalist, human nutrition, and basic research journals was also carried out. Two authors (DM and CP) independently reviewed the titles, abstracts, and full texts of the retrieved articles to determine their potential inclusion. Any disagreements were resolved via discussion with a third author (SC). Manuscripts concerning the role of dual and triple agonists were considered. This review aims to explore the role of dual and triple agonists, in the treatment of MASLD, highlighting from the pathophysiology and mechanisms of action to the recent evidence from clinical trials.

### 1.1 Incretins and glucagon

GLP-1 and GIP are the two major representative incretin hormones, secreted from the intestine in response to the presence of glucose and other nutrients contained in food (Koufakis et al., 2023). GIP is the primary incretin hormone secreted by K cells of the proximal small intestine whereas GLP1 is secreted by the L cells.

The glucagon-like peptide-1 receptor (GLP-1R) is a member of the secretin receptor (family B) of G protein–coupled receptors (GPCRs) and is expressed in pancreatic beta (β) cells and alpha (α) cells, in the gastrointestinal tract, in the heart, in kidney and in neurons of both the peripheral and central nervous systems.

GIP Receptor (GIPR) is expressed in pancreatic β-cells, in adipose tissue, in neurons (in many non-overlapping areas with GLP-1R) of the central nervous system. Through these actions, GIP increases lipoprotein lipase activity inducing fat accumulation and promoting healthy growth of white adipose tissue, and increases adiponectin levels, which exert insulin-sensitizing effects on adipocytes. GIP may also inhibit food intake and exert a strong anorexigenic effect that is additive to GLP-1R signaling (Naseralallah and Aboujabal, 2023; Min and Bain, 2021; Várkonyi et al., 2023).

Incretins act as physiological bifunctional blood glucose stabilizers. GLP-1 is glucagonostatic and inhibits glucagon secretion during hyperglycemic and normoglycemic states, but not during hypoglycemia. In contrast, GIP is both insulinotropic and glucagonotropic in a glucose-dependent manner, stimulating insulin secretion during hyperglycemia and glucagon secretion during hypoglycemia or fasting. GIP plays little to no role in gastric acid secretion or gastric emptying.

Plasma concentrations of GIP and GLP-1 are very low during fasting and increase within 15–30 min after meal ingestion. However, incretins have a short duration of action (approximately 1–2 min) because they are rapidly inactivated by the dipeptidyl peptidase-4 (DPP-4) enzyme (Min and Bain, 2021; Sardar et al., 2024).

Glucagon is a key regulator of metabolic homeostasis whose physiological role extends beyond its classical counter-regulatory action on insulin. In addition to stimulating hepatic glucose production, glucagon modulates lipid, amino acid, and energy metabolism. At the hepatic level, glucagon reduces triglyceride synthesis and very-low-density lipoprotein secretion, promotes fatty acid β-oxidation and ketogenesis, and limits hepatic lipid accumulation, contributing to improvements in steatosis and dyslipidaemia. Glucagon also plays a central role in postprandial amino acid handling and interacts with incretin hormones to coordinate nutrient disposal following food intake.

Beyond substrate metabolism, glucagon contributes to body-weight regulation by suppressing appetite, delaying gastric emptying, and increasing energy expenditure through indirect thermogenic mechanisms (Habegger et al., 2010). These effects are mediated primarily through the glucagon receptor (GCGR), a class B G protein–coupled receptor highly expressed in the liver and present in extrahepatic tissues, with downstream activation of cAMP-dependent signalling and additional Gq-mediated pathways implicated in lipid and energy homeostasis (Habegger et al., 2010).

This multifaceted biology provides a strong rationale for therapeutic strategies combining glucagon receptor activation with incretin signalling (Figure 1).

**FIGURE 1:**
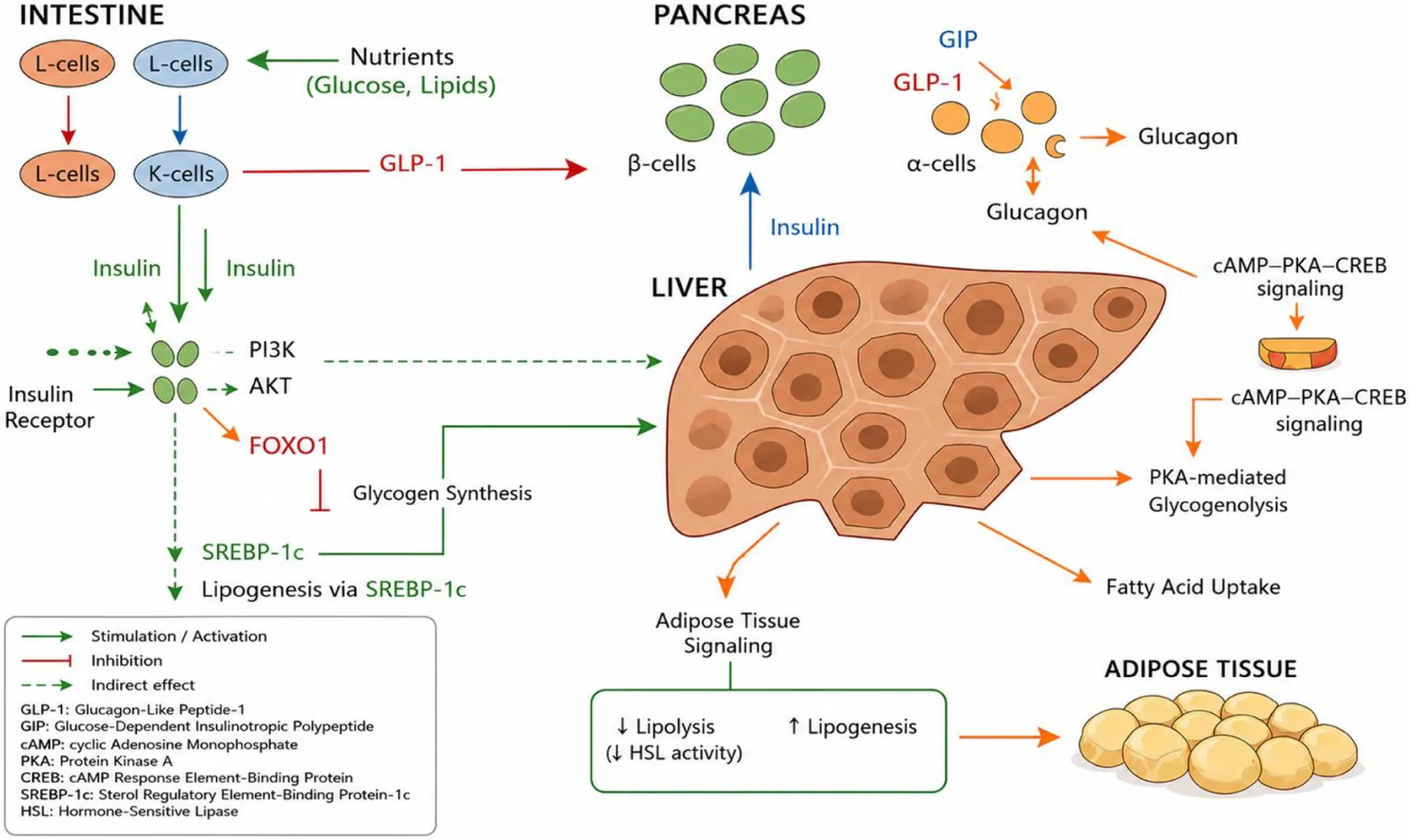
Integrated incretin–insulin–glucagon signaling pathways regulating hepatic glucose and lipid metabolism through the gut–pancreas–liver–adipose tissue axis. Following nutrient ingestion, intestinal enteroendocrine L-cells and K-cells secrete incretin hormones, including GLP-1 and GIP, which enhance glucose-stimulated insulin secretion from pancreatic β-cells and modulate glucagon release from α-cells. In hepatocytes, insulin signaling through the PI3K/AKT pathway promotes glycogen synthesis and regulates *de novo* lipogenesis via SREBP-1c while suppressing gluconeogenic transcriptional programs. Conversely, glucagon activates the hepatic cAMP–PKA–CREB signaling cascade, stimulating glycogenolysis and influencing fatty acid metabolism. Crosstalk between the liver and adipose tissue further modulates fatty acid uptake, lipogenesis, and lipolysis. The coordinated actions of incretin hormones, insulin, glucagon, and adipose tissue–derived signals are essential for maintaining systemic glucose and lipid homeostasis, whereas dysregulation of these pathways contributes to insulin resistance and the development and progression of metabolic dysfunction-associated steatotic liver disease (MASLD). Abbreviations: GLP-1, glucagon-like peptide-1; GIP, glucose-dependent insulinotropic polypeptide; PI3K, phosphoinositide 3-kinase; AKT, protein kinase B; SREBP-1c, sterol regulatory element-binding protein 1c; cAMP, cyclic adenosine monophosphate; PKA, protein kinase A; CREB, cAMP response element-binding protein; MASLD, metabolic dysfunction-associated steatotic liver disease.

## 2 Dual and triple agonists

The landscape of metabolic disease treatment, particularly for T2D, obesity, and MASLD has shifted from single-hormone mimics to multi-receptor agonists, particularly dual and triple agonists. (Sanyal et al., 2025).

These “nutrient-stimulated” peptides aim to achieve greater efficacy by targeting multiple metabolic pathways simultaneously. Beyond simple weight loss or glycemic control, these multi-agonists are showing incredible promise in treating the underlying metabolic syndrome as a whole. They actively work to reduce systemic inflammation, improve lipid profiles, and directly clear fat from the liver—a critical factor in treating MASLD. By simultaneously addressing the brain’s hunger signals, the pancreas’s insulin secretion, and the liver’s energy expenditure, these therapies are closing the gap between traditional medication and the life-altering results previously only seen with bariatric surgery. We are moving toward an era where “metabolic health” is treated as a unified system rather than a collection of separate diseases (Figure 2).

**FIGURE 2:**
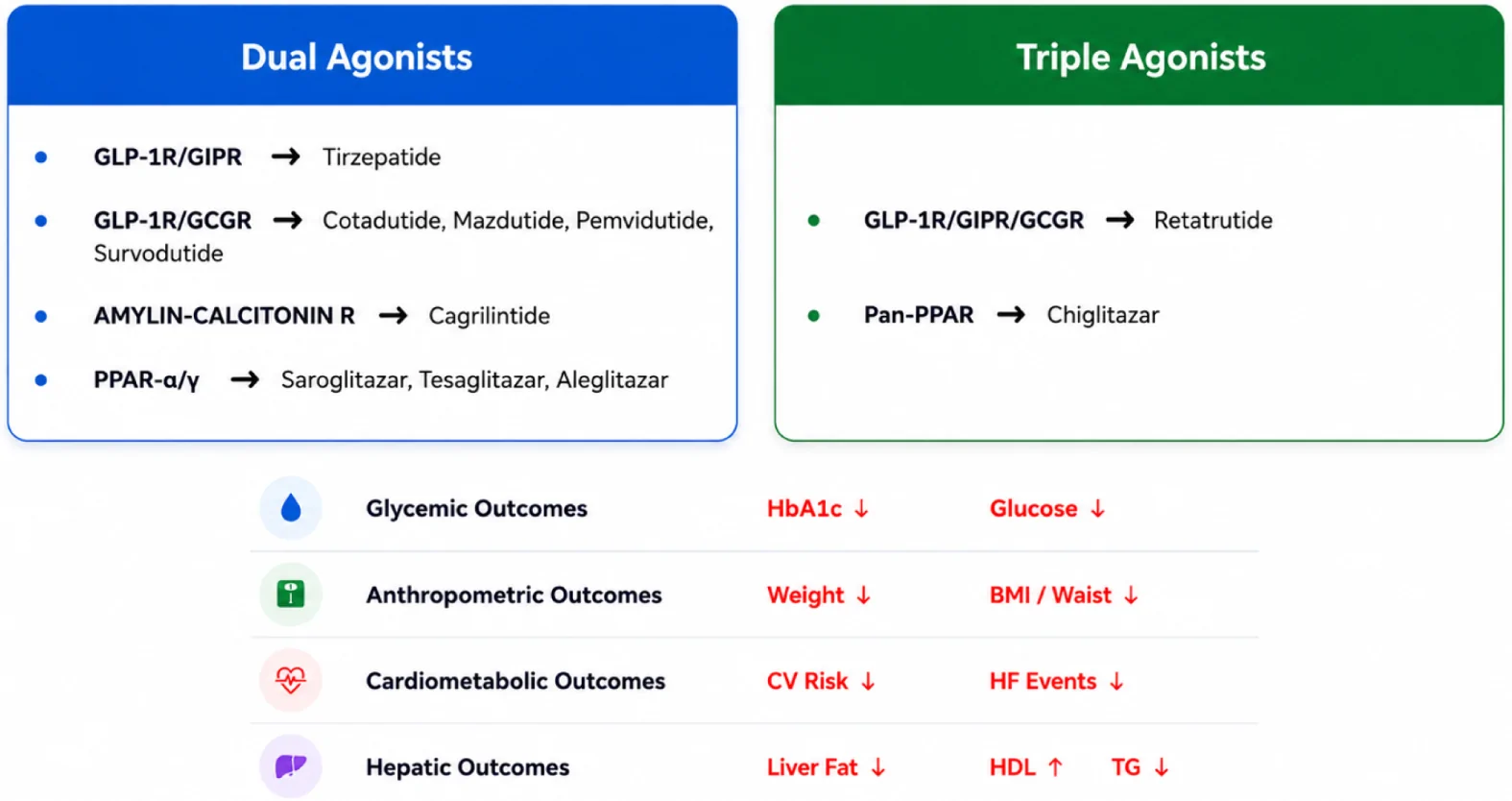
Overview of dual and triple agonists. Receptor combinations, main glycemic, anthropometric, cardiometabolic, and hepatic outcomes reported in clinical studies. Abbreviations: GLP-1R, glucagon-like peptide-1 receptor; GIPR, glucose-dependent insulinotropic polypeptide receptor; GCGR, glucagon receptor; PPAR, peroxisome proliferator–activated receptor; HbA1c, glycated hemoglobin; BMI, body mass index; CV, cardiovascular; HF, heart failure; HDL, high-density lipoprotein; TG, triglycerides.

## 3 Dual agonists

### 3.1 Epidemiology and pathophysiology of MASLD

MASLD, formerly classified as non-alcoholic fatty liver disease (NAFLD), is defined by the presence of hepatic steatosis in individuals exhibiting one or more cardiometabolic risk factors, in the absence of excessive alcohol intake (Tacke et al., 2024). MASLD represents a highly prevalent condition, affecting approximately 25%–38% of the global population and up to 70% of individuals with T2D, thereby constituting the most frequent chronic liver disease worldwide (Wong et al., 2023). Despite its epidemiological relevance, approved pharmacological therapies remain limited. MASLD is consistently associated with metabolic syndrome components, including IR, obesity, dyslipidemia, and hypertension (Eslam et al., 2020). Alterations in adipose tissue biology are central to disease pathogenesis. In obesity, expansion of adipose tissue mass is accompanied by adipocyte hypertrophy, hyperplasia, and immune cell infiltration, resulting in a pro-inflammatory microenvironment that contributes to systemic low-grade inflammation (Bertola et al., 2012; Luci et al., 2020). This inflammatory state promotes IR and enhanced lipolytic activity, leading to increased circulating non-esterified fatty acids (NEFAs) and subsequent hepatic lipid accumulation. Excessive fatty acid flux into hepatocytes promotes lipotoxicity and cellular injury, generating damage-associated molecular patterns (DAMPs). Obesity is further associated with gut microbiota alterations, increased intestinal permeability, and translocation of bacterial-derived pathogen-associated molecular patterns (PAMPs), including lipopolysaccharides (LPS), into the portal circulation. These signals, together with metabolic stress and hepatocyte death, contribute to sustained hepatic inflammation, progression to MASH, fibrogenesis, and increased risk of hepatocellular carcinoma (HCC). Both innate and adaptive immune cells, including myeloid and lymphoid populations, produce elevated levels of pro-inflammatory cytokines such as interleukin-1 (IL-1), IL-6, and tumor necrosis factor-α (TNF-α), while anti-inflammatory mediators, including IL-10, are reduced. This inflammatory milieu disrupts adipocyte function, resulting in impaired insulin signaling, increased lipolysis, and an altered adipokine profile characterized by increased pro-inflammatory and pro-fibrogenic mediators, such as leptin and osteopontin, alongside reduced insulin-sensitizing and anti-fibrogenic adipokines, including adiponectin and resistin. These alterations contribute to the development of systemic IR, T2D, and progression of MASLD.

### 3.2 Gut–liver axis and progression to MASH

The gut–liver axis plays an additional role, as intestinal dysbiosis in obesity is associated with reduced availability of choline and short-chain fatty acids, altered bile acid composition, and increased endogenous ethanol production. Hepatic lipid accumulation results from increased substrate delivery combined with impaired lipid oxidation and reduced export through very-low-density lipoproteins (VLDL). Fatty acids are initially esterified into triglycerides and stored within lipid droplets as a protective mechanism; however, excessive lipid burden overwhelms this process and promotes hepatocellular lipotoxicity (Luci et al., 2020). Lipotoxic injury is associated with persistent cellular stress responses, including oxidative and endoplasmic reticulum stress, and impaired autophagic flux, leading to accumulation of danger signals such as DAMPs, extracellular vesicles, and senescent hepatocytes, thereby facilitating disease progression (Gual et al., 2017). Hepatocytes, Kupffer cells, hepatic stellate cells, and sinusoidal endothelial cells respond to metabolic stress, DAMPs, and PAMPs, initiating and sustaining inflammatory signaling pathways (Luci et al., 2019). This process reshapes the hepatic immune environment through recruitment of additional immune cell populations, depletion of regulatory T cells, and enrichment of pro-inflammatory T-helper 1 (Th1) responses (Sutti and Albano, 2020). Consequently, hepatic cytokine profiles shift toward a pro-inflammatory state, characterized by reduced levels of IL-10 and transforming growth factor-β (TGF-β), and increased secretion of IL-1, IL-6, and TNF-α (Bourinet et al., 2024). Chronic inflammation promotes hepatocyte apoptosis, further amplifying DAMP release, while apoptotic bodies activate Kupffer cells and hepatic stellate cells, leading to increased extracellular matrix deposition, including type I collagen, and progression of fibrosis (Malhi and Gores, 2008). Persistent activation of these pathways ultimately results in cirrhosis and increased HCC risk.

### 3.3 GLP-1 receptor agonists and emerging incretin-based therapies

GLP-1RAs, including single and dual agonists such as GIP/GLP1-RAs, are approved for the management of T2D, with selected agents also indicated for obesity treatment, including liraglutide, semaglutide, and tirzepatide. These agents have demonstrated beneficial cardiovascular and renal effects (Newsome and Ambery, 2023). Their mechanisms include glucose-dependent enhancement of insulin secretion, appetite suppression, increased satiety, and delayed gastric emptying, resulting in clinically relevant weight loss (Drucker, 2018). Additional hormonal pathways involving GIP, glucagon, and amylin analogues, such as cagrilintide, have been leveraged to potentiate GLP-1–mediated effects and to induce complementary actions on lipid metabolism, energy expenditure, and adipose tissue function. These approaches have led to the development of dual and triple receptor co-agonists capable of inducing weight loss comparable to bariatric surgery (Nogueiras et al., 2023). Pharmacological agents currently under investigation for MASH include semaglutide; dual GLP-1/GIP agonists, such as tirzepatide; dual GLP-1/glucagon agonists, including cotadutide, survodutide, and efinopegdutide; and triple GLP-1/GIP/glucagon agonists, such as retatrutide. In the largest completed randomized trial, semaglutide achieved a significantly higher rate of steatohepatitis resolution compared with placebo over an 18-month treatment period, although no significant improvement in fibrosis was observed (Newsome et al., 2021). The use of semaglutide, has already demonstrated more consistent histological benefits in a broader population of patients with MASH and significant fibrosis. In the phase 3 ESSENCE trial, semaglutide 2.4 mg once weekly demonstrated significant benefits in patients with MASH: after 72 weeks of treatment, semaglutide led to a significantly higher rate of MASH resolution without worsening of fibrosis compared with placebo (62.9% vs. 34.3%), and a greater proportion of patients achieved fibrosis improvement without worsening of steatohepatitis (36.8% vs. 22.4%). These histological improvements were accompanied by substantial weight loss, supporting semaglutide as an effective therapeutic option for patients with MASLD/MASH and clinically significant liver fibrosis (Sanyal et al., 2025). A phase III registrational trial is ongoing, and combination strategies involving lipogenesis inhibitors are under evaluation (Loomba et al., 2021). Histological outcomes for newer dual and triple agonists are not yet available (Romero-Gómez et al., 2023). Tirzepatide has demonstrated substantial reductions in hepatic and visceral adiposity in individuals with T2D, accompanied by marked weight loss comparable to bariatric surgery, and preliminary phase II data suggest potential efficacy in steatohepatitis resolution. Dual GLP-1/glucagon agonists have also shown improvements in hepatic steatosis, liver enzyme levels, and non-invasive fibrosis markers in patients with MASLD. Weight reduction remains a key determinant of MASH outcomes (Rinella et al., 2023; Promrat et al., 2010).

### 3.4 Tirzepatide

Tirzepatide (LY3298176) is a 39–amino acid linear peptide, comparable in size to GLP-1 and GIP. Its amino acid sequence retains nine homologous residues from GLP-1 and ten amino acids shared between GIP and GLP-1. Four amino acids correspond to identical positions in the GLP-1 molecule, and ten amino-terminal amino acids are identical to those of exendin-4, a peptide derived from the saliva of the lizard *Heloderma suspectum* that inspired exenatide, the first GLP-1 receptor agonist. In addition, three amino acid positions are unique to tirzepatide, and amino-isobutyric acid residues are present at positions 2 (a DPP-4 recognition site) and 13. An (AEEA)2-gamma Glu-C20 diacid is attached to the lysine residue in position 20, which reduces degradation by DPP-4 and enhances binding to albumin; it’s also essential in increasing a long duration of action and allowing once-weekly dosing (like in GLP-1RAs semaglutide and liraglutide as well as in long-acting insulin analogues degludec and icodec). In pancreatic ß-cells, tirzepatide promotes cyclic Adenosine MonoPhosphate (cAMP) production in a greater sense than a combination of GIP and GLP-1 (Nauck and Tirzepatide, 2022).

Tirzepatide binds to and stimulates the GIPR with comparable affinity to GIP, but its affinity for the GLP-1R is about fivefold less strong than for GLP-1 (France and Syed, 2024).

This is also demonstrated by signaling studies: signaling studies have shown that tirzepatide behaves as a partial agonist at the GLP-1R (approximately 51% efficacy), preferentially promoting cAMP production over β-arrestin recruitment (low efficacy, <10% Emax), which may result in reduced GLP-1R internalization (Naseralallah and Aboujabal, 2023).

Accordingly, Tirzepatide leads to lower fasting and postprandial glycemia, decrease food intake, decreases glucagon levels and increases first and second-phase insulin production, both in glucose-dependent manner, in diabetic patients. Furthermore, tirzepatide has also been shown to increase adiponectin level.

The molecule is administered as a weekly subcutaneous (SC) injection (Al-Horani and Chedid, 2023) and the site of SC administration (abdomen, thigh or upper arm) does not interfere the pharmacokinetic properties; no drug interactions have been reported.

The tmax [time to maximum plasma concentration (Cmax)] of tirzepatide ranges from 8 to 72 h after subcutaneous administration, with a mean absolute bioavailability of 80%. Steady-state plasma concentrations are obtained after 4 weeks of once-weekly dosing, and its apparent volume of distribution at steady state is about 10.3 L. It’s also 99% bound to plasma albumin.

Tirzepatide is metabolised by beta (β)-oxidation of the C20 fatty acid moiety and hydrolysis of the amide and proteolytic cleavage of the peptide chain; metabolites are excreted in urine and faeces, and unmetabolised tirzepatide is not present in urine or faeces. Tirzepatide has an apparent mean plasma clearance of 0.061 L/h and a mean elimination half-life of ≈5 days. Its pharmacokinetics are not clinically significantly affected by sex, age, ethnicity, race, body weight, or renal or hepatic impairment, so the dosage of tirzepatide is unlikely to need to be modified in patients with chronic kidney disease or reduced hepatic function (France and Syed, 2024; Nauck and Tirzepatide, 2022). However, tirzepatide, due to the temporary delay in gastric emptying, may affect the absorption of concomitant oral medications, and this effect is greater after the first dose of the drug. For this reason, tirzepatide may affect the absorption of oral contraceptives. In this regard, it is worth to outline that the United States states people using oral contraceptives are recommended to switch to forms of contraception other than oral contraceptives for 4 weeks after the initial dose of tirzepatide and for 4 weeks after each dose increase; In contrast, EU does not recommend any adjustment of the dose of oral contraceptives because the interaction is not considered clinically relevant (France and Syed, 2024).

### 3.5 Clinical evidence

Clinical trials evaluating tirzepatide in individuals with overweight or obesity have demonstrated robust and dose-dependent reductions in body weight. In these studies, tirzepatide consistently achieved clinically meaningful weight loss, exceeding that observed with selective GLP-1 receptor agonists. These effects were observed both in the presence and absence of type 2 diabetes, supporting a direct role of tirzepatide in obesity treatment beyond glycemic control. In addition to body weight loss, tirzepatide treatment is associated with significant improvements in obesity-related metabolic abnormalities (enhanced insulin sensitivity, favorable changes in lipid profiles, and reductions in markers of metabolic inflammation) and comorbidities (Sattar et al., 2022; Wang et al., 2025).

It is worth to be outlined the SURMOUNT-1 trial (included in the SURMOUNT program), a phase 3, randomized, double-blind trial evaluating tirzepatide in adults with obesity or overweight without diabetes. In this trial, Tirzepatide induced substantial, dose-dependent weight loss over 72 weeks, with mean reductions approaching or exceeding 20% at higher doses. Importantly, a high proportion of participants achieved ≥15% and ≥20% weight loss, outcomes previously attainable primarily through bariatric surgery. Extended analyses demonstrated favorable effects on cardiometabolic risk factors (systolic blood pressure, dyastolic blood pressure, triglycerides and waist circumference) (Linetzky et al., 2025) and a reduced incidence of progression to T2D, showing significantly reduction of the predicted 10-year risk of atherosclerotic cardiovascular disease (ASCVD) compared to placebo—relative risk reduction ranged between ∼16 and 24%. This suggests that improvements in modifiable risk factors (blood pressure, lipids, glycaemia) could translate into lower future cardiovascular risk, although ongoing cardiovascular outcomes trials are needed to confirm effects on actual events.

In patients with T2D, tirzepatide therapy has been associated with reductions in liver fat content and improvements in biomarkers of MASH and fibrosis (Coskun et al., 2018).

Using data from the SURPASS-3 MRI substudy, Gastaldelli et al. (2022) demonstrated efficacy in steatohepatitis resolution, while effects on fibrosis regression remain modest. Moreover, Cariou et al. (2024) conducted an exploratory analysis evaluating the effects of tirzepatide on body fat distribution patterns independent of body mass index (BMI) in individuals with T2D. Fat distribution was characterized through sex- and BMI-adjusted z-scores for visceral adipose tissue (VAT), abdominal subcutaneous adipose tissue (aSAT), and liver fat (LF), derived by comparison with matched virtual control groups from the UK Biobank imaging cohort. At baseline, participants with T2D exhibited a pathological fat distribution phenotype, characterized by excess visceral adiposity and hepatic steatosis (elevated z-VAT and z-LF), alongside relatively reduced subcutaneous fat storage capacity (negative z-aSAT), compared with sex- and BMI-matched controls. Baseline fat distribution profiles were comparable between tirzepatide and insulin degludec treatment arms. After 52 weeks of treatment, Tirzepatide induced a significant and differential remodeling of fat depots, beyond what would be expected from weight loss alone. Specifically, tirzepatide treatment was associated with a significant reduction in visceral fat (Δz-VAT −0.18; p < 0.001), a marked decrease in liver fat (Δz-LF −0.54; p < 0.001) and a relative increase in abdominal subcutaneous fat (Δz-aSAT +0.11; p = 0.012). Importantly, these changes resulted in a convergence of all fat z-scores toward zero, indicating a shift toward a more balanced and metabolically favorable fat distribution pattern, with preferential loss of high-risk ectopic fat depots (VAT and LF) and relative preservation of subcutaneous adipose tissue. In contrast, insulin degludec treatment led to a significant reduction in liver fat only (Δz-LF −0.46; p = 0.001), while changes in visceral and subcutaneous fat distribution were not statistically significant, suggesting no meaningful redistribution of adiposity beyond hepatic fat reduction (Cariou et al., 2024).

The SYNERGY-NASH trial evaluated the efficacy and safety of tirzepatide in patients with biopsy-confirmed MASH and moderate-to-severe fibrosis. The 52 weeks trial showed that tirzepatide was highly effective and superior to the placebo. In particular, up to 73% of patients with stage 2 (F2) or stage 3 (F3) liver fibrosis and a high NAFLD activity score taking tirzepatide achieved resolution of MASH without their fibrosis worsening. In addition, they also showed significant improvement in the reduction of liver fibrosis staging. The trial demonstrated consistent benefits across patient subgroups, including those with Type 2 diabetes. It also resulted in reduced body weight, improved liver biochemistry, and decreased liver fat (Loomba et al., 2024).

### 3.6 Drug tolerability and safety

Beyond its efficacy, the safety and tolerability of tirzepatide warrant careful consideration. A *post hoc* analysis evaluated the relationship between gastrointestinal adverse events and weight loss in adults with obesity or overweight, with or without T2D, treated with tirzepatide across the four global Phase 3 SURMOUNT trials. The analysis focused on self-reported gastrointestinal adverse events—specifically nausea, vomiting, diarrhoea, and dyspepsia—and examined whether the occurrence of these symptoms contributed meaningfully to the magnitude of weight reduction observed with Tirzepatide treatment. Across SURMOUNT-1 through SURMOUNT-4, gastrointestinal adverse events were more frequently reported with tirzepatide than with placebo (27.8%–72.8% vs. 12.2%–32.5%, respectively) and occurred predominantly during the dose-escalation phase. Most gastrointestinal events were mild to moderate in severity, non-serious, and transient. Discontinuation of Tirzepatide due to gastrointestinal adverse events was uncommon, ranging from 1.0% to 10.5% across trials. Importantly, clinically meaningful weight reduction with tirzepatide was observed irrespective of the presence of gastrointestinal adverse events. Participants who reported no nausea, nausea alone, or any gastrointestinal symptom experienced comparable relative weight loss at the primary efficacy endpoints. Furthermore, longitudinal weight-loss trajectories over time were broadly similar between participants with and without gastrointestinal adverse events, indicating that gastrointestinal symptoms did not materially mediate or drive the weight-reducing effects of tirzepatide (Rubino et al., 2025).

Other adverse events include pancreatitis, cholecystitis, injection site reactions, hypersensitivity and antibody formation (Naseralallah and Aboujabal, 2023). Superior weight reduction with tirzepatide versus comparators appears to be independent of reported gastrointestinal events (Patel et al., 2024). A systematic review and meta-analysis suggested also the cardiovascular safety of tirzepatide as there was no increase in major adverse cardiovascular events (MACE) (Kaore et al., 2024). Tirzepatide is contraindicated in patients with a personal or family history of medullary thyroid carcinoma and in patients with multiple endocrine neoplasia syndrome type 2; moreover, tirzepatide delays gastric emptying, which may reduce the effectiveness of oral contraceptives, so if switching to a non–oral contraceptive method is not preferred by the patient, a barrier method is recommended for 4 weeks after initiation and dose escalation (Rebitch, 2023).

### 3.7 Mechanisms of action and future perspectives

GLP-1Rs are expressed across multiple organs, including the liver, pancreas, gastrointestinal tract, central nervous system, and cardiovascular system, supporting metabolic effects beyond glycemic regulation (Ludvik et al., 2021). Activation of GLP-1 signaling improves insulin sensitivity, suppresses hepatic gluconeogenesis, enhances fatty acid oxidation, and reduces *de novo* lipogenesis through modulation of transcriptional regulators such as sterol regulatory element-binding protein-1c (SREBP-1c) and carbohydrate-responsive element-binding protein (ChREBP), while also exerting anti-inflammatory effects by reducing circulating TNF-α, IL-6, and C-reactive protein levels (Armstrong et al., 2016). Meta-analyses evaluating dual agonist therapies in MASLD have reported significant reductions in hepatic steatosis compared with control interventions, likely reflecting complementary mechanisms whereby GLP-1R activation primarily improves insulin sensitivity and hepatic glucose metabolism, while GIP receptor signaling enhances lipid handling and adipose tissue function (Fisman and Tenenbaum, 2021; Regmi et al., 2024). Current management of MASLD remains focused on lifestyle modification, weight reduction, and optimization of metabolic comorbidities. The 2024 approval by the Food and Drug Administration of resmetirom as the first targeted pharmacological therapy for MASH with liver fibrosis represents an important advancement; however, treatment availability remains limited and long-term outcome data are still emerging (Alshehade, 2024). Effective pharmacological options remain unavailable for most patients with MASLD, particularly those with non-cirrhotic disease, despite recent advances in the treatment of MASH with fibrosis. (Chalasani et al., 2018).

### 3.8 GLP-1R/GCGR dual agonists

Dual agonism targeting the GLP-1R and the GCGR aims to combine complementary metabolic actions within a single therapeutic molecule. GLP-1 receptor activation improves glycaemic control and reduces appetite, whereas glucagon receptor activation increases energy expenditure and modulates hepatic lipid metabolism. The rationale of this approach is to enhance weight loss and metabolic flexibility beyond that achievable with GLP-1RA alone, while relying on GLP-1 signalling to counterbalance the hyperglycaemic effects of glucagon.

Since GLP-1 and glucagon are proglucagon-derived peptides with coordinated physiological roles, their combined pharmacological targeting is biologically plausible. However, pharmacological glucagon receptor activation requires a finely tuned balance to preserve glycaemic stability, making dual agonism inherently compound-specific rather than a uniform class effect (Sandoval and D’Alessio, 2015; Novikoff et al., 2021; Winther and Holst, 2024).

#### 3.8.1 Cotadutide

Cotadutide is a first-generation unimolecular GLP-1/glucagon dual agonist engineered to favour GLP-1 receptor agonism while retaining biologically relevant glucagon receptor activity. This conservative receptor balance was intentionally designed to limit glucagon-induced hyperglycaemia while preserving potential metabolic benefits. Compared with later-generation compounds, cotadutide reflects an early design strategy prioritising glycaemic stability and tolerability over maximal metabolic efficacy (Boland et al., 2020; Nahra et al., 2021).

##### 3.8.1.1 Randomized controlled trials (RCTs)

In a 54-week randomised phase 2b trial in adults with overweight or obesity and T2D, cotadutide induced modest but statistically significant reductions in body weight and HbA1c (Roth et al., 2020), alongside improvements in selected metabolic and hepatic parameters. Although these findings established clinical feasibility of dual GLP-1/glucagon agonism, the magnitude of weight loss and glycaemic improvement was limited compared with newer incretin-based therapies (Nahra et al., 2021).

##### 3.8.1.2 Post hoc analyses and mechanistic studies

Mechanistic studies in humans confirmed biologically relevant glucagon receptor engagement with cotadutide, including stimulation of hepatic glycogenolysis (Parker et al., 2023). These findings provided direct evidence of GCGR activation *in vivo* while underscoring the need for careful receptor balancing to preserve glycaemic control.

##### 3.8.1.3 Smaller studies and preclinical evidence

Clinical evidence for cotadutide is primarily derived from a single long-duration phase 2b trial, which, while sufficient to establish feasibility, limits the available comparative data on efficacy. Preclinical data also suggested effects on hepatic lipid metabolism (Boland et al., 2020), although translation to robust clinical outcomes was limited.

##### 3.8.1.4 Safety and tolerability

Cotadutide was generally well tolerated, with gastrointestinal adverse events representing the most frequently reported side effects and occurring predominantly during treatment initiation. No major safety signals or clinically relevant deterioration in glycaemic control were observed during prolonged exposure. However, the need to suppress glucagon receptor activity to maintain metabolic safety has likely contributed to the modest efficacy profile observed in clinical trials (Nahra et al., 2021; Parker et al., 2023).

#### 3.8.2 Mazdutide

Mazdutide is a long-acting unimolecular GLP-1/glucagon dual agonist developed to achieve sustained receptor engagement and a more effective balance between metabolic efficacy and glycaemic safety compared with first-generation compounds. Its molecular design allows greater glucagon receptor activation than cotadutide, relying on concomitant GLP-1RA and prolonged exposure to mitigate hyperglycaemic risk (Ji et al., 2023; Zhang et al., 2024).

##### 3.8.2.1 Randomized controlled trials (RCTs)

In a phase 2 randomised controlled trial conducted in adults with overweight or obesity, once-weekly mazdutide induced clinically meaningful and dose-dependent reductions in body weight compared with placebo (Ji et al., 2023), establishing robust metabolic efficacy in a non-diabetic population.

In patients with T2D, a randomised, double-blind, placebo-controlled phase 2 trial demonstrated that mazdutide significantly reduced HbA1c while also promoting weight loss (Ji et al., 2023; Zhang et al., 2024), without evidence of glucagon-mediated deterioration of glycaemic control.

##### 3.8.2.2 Systematic reviews and meta-analyses

The consistency of these findings across randomised trials was further supported by a recent systematic review and meta-analysis, which confirmed significant improvements in body weight and glycaemic outcomes (Nalisa et al., 2024) with mazdutide while highlighting the limited duration of available studies.

##### 3.8.2.3 Safety and tolerability

Mazdutide exhibited a safety profile largely consistent with GLP-1 receptor agonists. Gastrointestinal adverse events were the most frequently reported and were generally dose-dependent and transient. Despite enhanced glucagon receptor engagement, no major glycaemic safety signals were observed in short- to medium-term studies, supporting an acceptable benefit-risk profile at investigated doses (Ji et al., 2023; Zhang et al., 2024; Nalisa et al., 2024).

#### 3.8.3 Pemvidutide

Pemvidutide is a long-acting unimolecular GLP-1/glucagon dual agonist developed with sustained pharmacokinetic exposure and a receptor balance suitable for chronic treatment. In contrast to earlier dual agonists primarily focused on weight and glycaemic control, pemvidutide was advanced with a specific interest in hepatic metabolic effects, reflecting a strategic shift toward organ-targeted applications of dual agonism (Harrison et al., 2025; Browne et al., 2025).

##### 3.8.3.1 Randomized controlled trials (RCTs)

Clinical evidence for pemvidutide derives from phase 2 randomised, placebo-controlled trials primarily conducted in patients with MASLD and MASH. In a 24-week randomised controlled study in patients with MASLD, weekly pemvidutide treatment resulted in significant reductions in liver fat content (Harrison et al., 2025), alongside improvements in metabolic biomarkers and body weight compared with placebo.

Consistent hepatic efficacy was subsequently confirmed in the multicentre, randomised, double-blind phase 2b IMPACT trial in patients with MASH. In this study, pemvidutide induced clinically meaningful improvements in liver-related endpoints over 24 weeks (Noureddin et al., 2025), supporting a potential disease-modifying effect in metabolic liver disease.

##### 3.8.3.2 Smaller studies and supportive evidence

Additional controlled clinical data further reinforced the reproducibility of hepatic benefits across MASLD populations (Browne et al., 2025).

##### 3.8.3.3 Safety and tolerability

Across clinical studies, pemvidutide was generally well tolerated, with gastrointestinal adverse events being the most frequently reported side effects. Importantly, no clinically meaningful hepatotoxicity or worsening of liver function parameters was observed in patients with MASLD or MASH, and glycaemic control remained stable despite glucagon receptor engagement (Harrison et al., 2025; Browne et al., 2025; Noureddin et al., 2025).

#### 3.8.4 Survodutide

Survodutide is a once-weekly, later-generation unimolecular GLP-1/glucagon dual agonist designed to optimise the balance between GLP-1-mediated appetite suppression and glycaemic control and glucagon-mediated increases in energy expenditure. Compared with earlier dual agonists, survodutide exhibits a more assertive glucagon receptor engagement while maintaining metabolic safety through concomitant GLP-1 receptor activation and prolonged pharmacokinetic exposure (Le Roux et al., 2024).

##### 3.8.4.1 Randomized controlled trials (RCTs)

In a randomised, double-blind, placebo-controlled, dose-finding phase 2 trial in adults with obesity, once-weekly survodutide induced robust and dose-dependent reductions in body weight over 46 weeks, establishing high metabolic efficacy within the dual agonist class (Le Roux et al., 2024).

In people with T2D, a randomised clinical trial comparing survodutide with placebo and open-label semaglutide demonstrated dose-dependent reductions in HbA1c and body weight, with survodutide achieving greater weight loss at higher doses while maintaining clinically meaningful glycaemic improvement (Blüher et al., 2024).

##### 3.8.4.2 Post hoc analyses

Post hoc analyses further confirmed consistent efficacy across sex and baseline BMI subgroups, supporting broad applicability in heterogeneous patient populations. Beyond glycaemic and weight outcomes, survodutide was associated with reductions in systolic blood pressure in adults with obesity, as demonstrated in *post hoc* analyses of phase 2 trial data (Le Roux et al., 2024).

##### 3.8.4.3 Smaller studies and supportive evidence

Clinical studies also explored survodutide in populations with advanced metabolic liver disease, including compensated cirrhosis, where treatment was associated with favourable pharmacokinetics and metabolic effects without evidence of hepatic decompensation (Lawitz et al., 2024).

##### 3.8.4.4 Safety and tolerability

Across phase 2 clinical trials, survodutide was generally well tolerated. Gastrointestinal adverse events, including nausea, vomiting, and diarrhoea, were the most frequently reported and were predominantly dose-related and transient. Importantly, no major glycaemic safety concerns or clinically relevant hepatotoxicity were observed, including in patients with underlying liver disease. However, available safety data are derived from phase 2 trials of limited duration, and longer-term exposure will be required to fully characterise rare or delayed adverse events (Le Roux et al., 2024; Blüher et al., 2024; Lawitz et al., 2024).

### 3.9 Amylin and calcitonin

Amylin and calcitonin are peptide hormones involved in the regulation of energy balance, glucose homeostasis, and appetite control, and have increasingly been investigated as pharmacological targets in metabolic disorders. Amylin is a 37–amino acid peptide released from pancreatic β-cells together with insulin following nutrient ingestion and exerts its effects through both central and peripheral pathways, contributing to the regulation of postprandial glycemia by delaying gastric emptying, inhibiting glucagon secretion, and inducing satiety. Under physiological conditions, amylin acts synergistically with insulin; however, in insulin-resistant states and in T2D, alterations in amylin secretion and signaling occur, favoring impaired glycemic regulation and increased caloric intake (Young, 1997). Calcitonin, classically associated with calcium and bone metabolism, displays structural and functional similarities with amylin and signals through receptor complexes composed of the calcitonin receptor and receptor activity–modifying proteins (Hay et al., 2006). Both peptides exert anorexigenic actions by engaging central nervous system circuits, particularly within the area postrema and hypothalamic nuclei, resulting in reduced food consumption and body weight. The therapeutic relevance of amylin signaling was first demonstrated with pramlintide, a synthetic amylin analogue approved for the treatment of diabetes, which showed consistent, albeit modest, effects on body weight reduction and postprandial glucose control (Lutz, 2009).Nevertheless, the widespread clinical use of early amylin analogues was constrained by gastrointestinal adverse events and the need for multiple daily injections (Ratner et al., 2004). Advances in peptide design have enabled the development of long-acting amylin receptor agonists and dual amylin–calcitonin receptor agonists (DACRAs), aimed at improving metabolic efficacy, tolerability, and pharmacokinetic properties (Mack et al., 2010). Dual agonism targeting both amylin and calcitonin receptors has emerged as an attractive strategy due to the complementary and partially overlapping biological actions of these pathways. Pramlintide is a Food and Drug Administration (FDA)-approved amylin analogue developed as an adjunct to mealtime insulin therapy in individuals with type 1 diabetes or insulin-treated T2D (Ryan et al., 2008) While activation of the amylin receptor predominantly regulates appetite, gastric emptying, and glucagon suppression, engagement of the calcitonin receptor appears to amplify and prolong central satiety signals, potentially through distinct neuronal populations (Boyle et al., 2018).Preclinical investigations have shown that DACRAs produce greater and more sustained reductions in food intake and body weight than selective amylin receptor agonists, together with improvements in insulin sensitivity and lipid metabolism (Larsen et al., 2021). Pramlintide exhibits pharmacological properties similar to endogenous human amylin but with enhanced molecular stability, rendering it suitable for subcutaneous administration (Young, 1997). Despite this improvement, pramlintide remains a short-acting peptide, with a plasma half-life of approximately 20–45 min in humans, necessitating administration with each meal to effectively attenuate postprandial glucose excursions (Colburn et al., 1996). The improved stability of pramlintide relative to native human amylin was achieved by introducing three proline substitutions at positions 25, 28, and 29, inspired by the lower aggregation propensity of rat amylin (Hay et al., 2015). Beyond its effects on postprandial glycemia, pramlintide has demonstrated weight-reducing properties in several clinical studies (Whitehouse et al., 2002). Across multiple patient populations, including individuals with obesity and T2D who are at increased risk of hepatic steatosis, pramlintide therapy has been associated with body weight reductions ranging from approximately 2%–6%, supporting the potential role of amylin-based pharmacology in the management of obesity and non-alcoholic fatty liver disease. In experimental models of obesity and metabolic dysfunction, dual amylin–calcitonin receptor agonists have been shown to decrease adiposity, ameliorate hepatic steatosis, and reduce obesity-related inflammation, indicating benefits that extend beyond weight loss alone (Hjuler et al., 2016). These findings are particularly relevant in the context of MASLD. By lowering energy intake and improving metabolic efficiency, therapies based on amylin signaling may indirectly mitigate hepatic lipid accumulation and lipotoxic stress (Smits and Van Raalte, 2021). Clinical studies assessing next-generation amylin analogues and DACRAs, including cagrilintide, have reported marked weight loss in individuals with obesity, especially when administered in combination with GLP-1RAs (Lau et al., 2021) The rationale for combining amylin or calcitonin receptor agonism with incretin-based therapies is supported by their complementary mechanisms of action: GLP-1RAs primarily enhance insulin secretion and satiety via gut–brain communication, whereas amylin-based agents further suppress appetite, slow gastric emptying, and inhibit glucagon release, resulting in additive or synergistic effects on weight reduction (Dutta et al., 2024) Randomized clinical trials evaluating the co-administration of cagrilintide and semaglutide have demonstrated weight loss approaching that observed following bariatric surgery, underscoring the therapeutic potential of multi-hormonal strategies (Wilding et al., 2021). In addition to weight reduction, emerging data suggest that amylin and DACRAs may exert direct effects on lipid metabolism, inflammatory pathways, and energy expenditure, with possible implications for cardiometabolic risk reduction (Larsen et al., 2001). Although liver-specific outcome data remain limited, the substantial weight loss and improvements in insulin sensitivity achieved with these agents are expected to translate into secondary benefits for hepatic steatosis and metabolic liver disease (Dahl et al., 2022). Overall, dual agonism involving amylin and calcitonin receptors represents a rapidly advancing therapeutic approach with significant promise for the treatment of obesity and associated metabolic disorders. Ongoing and future studies will be required to define long-term efficacy, safety, and organ-specific outcomes, as well as the optimal role of these agents within combination regimens targeting multiple hormonal pathways (Ludvik et al., 2021).

## 4 Triple agonists

### 4.1 GLP-1R/GIPR/GCGR triple agonists

#### 4.1.1 Retatrutide

On the basis of this integrated hormonal framework, triple receptor agonism has emerged as a rational approach to simultaneously target appetite regulation, glucose homeostasis, and energy expenditure.

Retatrutide (LY3437943) is a unimolecular peptide agonist of the GIPR, GLP-1R, and glucagon receptor, developed to exploit the complementary actions of these pathways (Kaur and Misra, 2024). The molecule is derived from a GIP-based scaffold and incorporates non-proteinogenic amino acids to enhance stability and receptor balance, including α-aminoisobutyric acid to confer resistance to dipeptidyl peptidase-4 degradation and substitutions that enhance GCGR activity. Acylation with a C20 fatty diacid promotes reversible albumin binding, resulting in a terminal half-life of approximately 6 days and enabling once-weekly administration. *In vitro* and *in vivo* profiling demonstrates that Retatrutide exhibits higher potency at the GIP receptor, with balanced but biologically meaningful activity at the GLP-1 and glucagon receptors, a profile associated with maximal weight-loss efficacy while preserving glycaemic control (Coskun et al., 2022).

##### 4.1.1.1 Randomized controlled trials (RCTs)

More pronounced weight loss has been reported in individual long-duration phase II trials. In a randomised, double-blind study including 338 adults with obesity or overweight, Kanu et al. reported that once-weekly Retatrutide administered at doses of 1–12 mg resulted in mean weight reductions of 7.2%–17.9% at Week 24% and 8.7%–24.2% at Week 48, with the largest reductions observed at the 12 mg dose (Kanu et al., 2025a).

In a phase II randomised trial enrolling 338 participants with T2D treated for 36 weeks, Rosenstock et al. showed that Retatrutide at doses of 8 mg and 12 mg once weekly reduced HbA1c by more than 2.0 percentage points and body weight by approximately 15%–17%, outperforming both placebo and dulaglutide 1.5 mg. Additional improvements were observed in systolic blood pressure, triglycerides, and non-HDL cholesterol (Rosenstock et al., 2023).

Detailed analyses of body composition further indicate that Retatrutide-induced weight loss is predominantly driven by reductions in adipose tissue. In a DXA substudy involving 281 adults with T2D treated for 36 weeks, Coskun et al. (2025) demonstrated that reductions in total and visceral fat mass accounted for the majority of weight loss, while absolute lean mass declined modestly and the relative proportion of lean mass was largely preserved.

Retatrutide also exerts consistent effects on appetite regulation and eating behaviour. In a phase II trial involving 275 adults with T2D treated for 36 weeks, Kanu et al. (2025b) reported that Retatrutide at doses ≥4 mg significantly reduced perceived hunger, appetite, and disinhibition, with the most pronounced effects observed at 8 mg and 12 mg, alongside weight loss and HbA1c reductions exceeding those observed with placebo and dulaglutide.

In a randomized, double-blind, placebo-controlled phase 2a trial, Sanyal et al. evaluated the metabolic effects of Retatrutide in participants with obesity and MASLD. Once-weekly administration of Retatrutide for up to 48 weeks resulted in robust, dose-dependent reductions in liver fat content, with mean relative decreases at 24 weeks ranging from approximately 43% at the lowest dose to over 80% at higher doses, while placebo showed no meaningful change. Resolution of hepatic steatosis, defined as liver fat content <5%, was achieved in most participants receiving the highest doses. Reductions in liver fat were strongly associated with substantial weight loss, decreases in visceral and subcutaneous adipose tissue, and improvements in insulin sensitivity and lipid metabolism. Near-maximal hepatic fat reduction was observed at approximately 20% body-weight loss. Retatrutide treatment was also associated with favorable changes in biomarkers of hepatocellular injury and fibrogenesis, including significant reductions in cytokeratin-18 and pro-collagen type III (pro-C3), suggesting potential benefits beyond steatosis (Sanyal et al., 2024a).

##### 4.1.1.2 Systematic reviews and meta-analyses

The metabolic efficacy observed in preclinical models has been confirmed in phase II clinical trials. In a systematic review and meta-analysis by Abouelmagd et al. pooling data from three randomised controlled trials including 878 participants, once-weekly Retatrutide produced statistically significant, dose-dependent reductions in body weight, body mass index, and waist circumference compared with placebo over follow-up periods of 24–36 weeks, with the greatest effects observed at the 8 mg and 12 mg doses. Treatment was also associated with significant improvements in glycaemic control and blood pressure, and a substantially higher likelihood of achieving clinically meaningful weight-loss thresholds (≥5%, ≥10%, and ≥15%) (Abdrabou Abouelmagd et al., 2025).

Similarly, Tewari et al., analysing four randomised controlled trials, confirmed the superiority of Retatrutide over placebo for weight reduction and reported a clear dose-response relationship, with maximal efficacy observed at the 12 mg once-weekly dose (Tewari et al., 2025).

##### 4.1.1.3 Post hoc analyses

In a complementary analysis conducted in 338 adults with obesity or overweight treated for 48 weeks, Kanu et al. demonstrated that Retatrutide-induced weight loss was accompanied by significant reductions in perceived hunger and disinhibition, whereas cognitive restraint did not differ significantly from placebo, despite substantial weight loss (Kanu et al., 2025a). In both populations, changes in eating behaviour were significantly correlated with the magnitude of weight reduction.

In a *post hoc* exploratory analysis, Wen et al. (2025) investigated the mechanisms underlying the lipid-lowering effects of Retatrutide, with a specific focus on circulating ANGPTL3/8, in 251 adults with T2D and 309 adults with obesity or overweight without diabetes. Retatrutide induced dose-dependent reductions in circulating ANGPTL3/8 that paralleled improvements in serum triglycerides and LDL cholesterol and were significantly correlated with changes in body weight, BMI, and markers of insulin resistance. Complementary experiments in primary human hepatocytes demonstrated that Retatrutide and glucagon directly suppressed ANGPTL3/8 secretion via glucagon receptor activation, providing mechanistic support for a GCGR-mediated pathway contributing to the lipid benefits of Retatrutide (Wen et al., 2025).

##### 4.1.1.4 Preclinical and mechanistic evidence

Preclinical studies show that triple agonism induces greater reductions in body weight and adiposity than mono- or dual-agonist therapies through combined effects on food intake, gastric emptying, energy expenditure, lipid metabolism, and hepatic steatosis (Katsi et al., 2025).

Its action appears to be dose-dependent. It is metabolized by the liver, with no interaction with cytochrome P450s, with a reduction in drug-drug interaction (Ray, 2023).

##### 4.1.1.5 Safety and tolerability

Retatrutide exhibits a safety profile largely characterized by gastrointestinal adverse events that are dose-related and generally manageable with gradual dose escalation (Katsi et al., 2025).

In the meta-analysis by Abdrabou Abouelmagd et al. (2025), the safety profile was considered acceptable, with predominantly dose-related gastrointestinal adverse events including reduced appetite, nausea, vomiting, diarrhea, and constipation, consistent with incretin-based therapies.

Similarly, Tewari et al. (2025) reported that the adverse event profile of Retatrutide was generally comparable to that of control groups, with no significant safety signals identified, although gastrointestinal events remained among the most frequently reported adverse effects.

In the MASLD phase 2a trial, treatment was generally well tolerated, with transient gastrointestinal adverse events being the most commonly reported side effects and no evidence of hepatotoxicity throughout the study period (Sanyal et al., 2024a).

##### 4.1.1.6 Ongoing studies

Ongoing phase III TRIUMPH trials will be critical to establish long-term efficacy, cardiovascular safety, and durability of these effects across obesity, T2D, and obesity-related complications (Giblin et al., 2026).

#### 4.1.2 Efocipegtrutide

Efocipegtrutide (also referred to as HM15211) is a long-acting triple agonist targeting the GLP-1, GIP, and glucagon receptors and has shown promising therapeutic activity in obesity, with potential benefits in non-alcoholic steatohepatitis and hepatic fibrosis (Zhang et al., 2025).

#### 4.1.3 Ongoing studies

Efocipegtrutide is currently in phase 2 development for MASH. An ongoing randomized, placebo-controlled trial is evaluating histological resolution of steatohepatitis without worsening of fibrosis, together with effects on liver fat content, fibrosis markers, body weight, and metabolic parameters, using an adaptive MRI-PDFF-guided dose-selection design.

## 5 Peroxisome proliferator-activated receptors–PPARs

Peroxisome proliferator-activated receptors (PPARs) are ligand-responsive transcription factors belonging to the nuclear hormone receptor superfamily (Desvergne and Wahli, 1999). Upon engagement by endogenous or synthetic ligands—including fatty acids, their metabolic derivatives, and pharmacological agonists—PPARs orchestrate the transcriptional regulation of a broad repertoire of genes involved in lipid handling, glucose homeostasis, and adipogenic differentiation. Accordingly, PPARs have garnered considerable attention as pharmacological targets for the treatment of metabolic syndrome and its associated comorbidities (Wright et al., 2014; Pirat et al., 2012; Wang, 2010; Tobin and Freedman, 2006). To date, three PPAR isoforms have been characterized: PPARα, PPARγ, and PPARδ (also referred to as PPARβ). PPARα is predominantly expressed in tissues exhibiting high rates of fatty acid oxidation, such as the liver and brown adipose tissue, and pharmacological activation of this receptor by fibrates (e.g., bezafibrate and fenofibrate) has been widely exploited in the clinical management of dyslipidemia and hyperglycemia. PPARγ is primarily localized in adipose tissue, where it serves as a master regulator of adipocyte differentiation and lipid storage; consequently, PPARγ agonists, including thiazolidinediones such as pioglitazone, are approved for the treatment of T2D. In contrast, PPARδ displays a more ubiquitous expression profile and plays a pivotal role in fatty acid oxidation, energy expenditure, and reverse cholesterol transport. Notably, the selective PPARδ agonist GW501516 has been shown to improve insulin sensitivity and metabolic flexibility in murine models of obesity and T2D (Tanaka et al., 2003; Lee et al., 2006; Billin, 2008). In light of these pleiotropic metabolic functions, substantial efforts have been devoted to the development of PPAR-targeted ligands, encompassing pan-agonists, dual and subtype-selective agonists, as well as partial agonists and selective PPAR modulators (SPPARMs), with the overarching objective of maximizing therapeutic efficacy while mitigating mechanism-based adverse effects (Pirat et al., 2012). From a structural perspective, the vast majority of reported PPAR agonists incorporate either a carboxylic acid (CA) or a thiazolidinedione (TZD) pharmacophore, which is critical for receptor activation. These acidic head groups engage in specific interactions with key amino acid residues located within the activation function-2 (AF-2) helix, thereby stabilizing the ligand-binding domain in an active conformation that facilitates co-activator recruitment. To date, only a limited number of PPAR agonists devoid of CA or TZD moieties have been described, including the endogenous lipid mediator N-oleoylethanolamide (OEA) and benzenesulfonamide-based compounds such as INT131 (Fu et al., 2003; Higgins and Mantzoros, 2008; Motani et al., 2009). OEA exhibits agonistic activity toward PPARα and PPARδ, with EC_50_ values of 0.12 μM and 1.1 μM, respectively, whereas INT131 acts as a selective PPARγ modulator with a reported Ki of 10 nM (Okazaki et al., 2016). Metabolic syndrome, a disorder of lipid homeostasis characterized by aberrant lipid deposition, dyslipidemia, and visceral adiposity, markedly elevates the risk of cardiovascular and cerebrovascular morbidity. Its global prevalence currently encompasses roughly one-quarter of the population and continues to escalate, driven in large part by excessive dietary sugar consumption and adverse effects of certain pharmacotherapies. Consequently, the identification of novel bioactive compounds from natural sources with potential therapeutic or nutraceutical utility has emerged as a pressing priority (Tonstad and Després, 2011; Silveira Rossi et al., 2022; Saklayen, 2018; Chen et al., 2019; Xing et al., 2019).

### 5.1 Rubusoside

#### 5.1.1 Preclinical evidence

Among such candidates, rubusoside—a diterpene glycoside of the ent-kaurane class predominantly isolated from Rubus chingii var. suavissimus or Rubus suavissimus (Chinese sweet leaf tea)—has attracted considerable attention due to its exceptional sweetness and lipid-modulating properties. With a sweetness intensity approximately 114-fold greater than sucrose and low caloric content, rubusoside is widely employed as a natural sweetener and as a solubilizing agent for hydrophobic pharmaceuticals. Beyond its organoleptic applications, rubusoside has been shown to mitigate dyslipidemia, attenuate high-fat diet-induced weight gain, and exert antidiabetic effects, mediated at least in part through inhibition of Glut5/Glut1 transporters and α-glucosidase activity. Despite these promising bioactivities, the mechanistic basis of rubusoside’s regulation of lipid metabolism remains incompletely defined (Zhang et al., 2020; Jiang et al., 2024; Chen et al., 2020; Li et al., 2020; Myint et al., 2020; George Thompson et al., 2015). It was demonstrated that Rubus suavissimus alleviates high-fat diet-induced lipid dysregulation in Syrian golden hamsters via activation of peroxisome proliferator-activated receptors α and γ (PPARα/γ) and enhances adipogenesis in 3T3-L1 preadipocytes, suggesting that rubusoside—the predominant bioactive constituent—may act through analogous molecular pathways (Jiang et al., 2021; Koh et al., 2011). PPARα complements these functions by orchestrating fatty acid oxidation and broader aspects of lipid metabolism. Additional modulators, including galectin-3 (Lgals3) and mitogen-activated protein kinase-interacting serine/threonine kinase-2 (Mknk2), are upregulated in obesity and contribute to metabolic derangements; targeting Lgals3 has emerged as a promising intervention for metabolic syndrome, while Mknk2 inhibition confers protection against diet-induced obesity. Collectively, these insights underscore the importance of investigating whether rubusoside exerts its lipid-lowering and metabolic regulatory effects through coordinated modulation of PPARα/γ, Lgals3, and Mknk2 signaling networks (Zhao et al., 2020; Liu et al., 2021; Menini et al., 2016; Baek et al., 2015; Sandeman et al., 2020).

#### 5.1.2 Clinical evidence

To date, robust randomized clinical trials evaluating rubusoside in patients with MASLD or T2D are lacking. Consequently, its therapeutic potential remains largely supported by mechanistic and animal data.

#### 5.1.3 Drug tolerability and safety

The safety profile of rubusoside has primarily been evaluated in preclinical studies and in its widespread use as a natural sweetener. Available evidence suggests low toxicity and good tolerability at commonly used doses, with no major safety signals identified. However, robust clinical trials specifically assessing long-term administration in patients with MASLD or T2D are currently lacking. Consequently, the incidence of clinically relevant adverse events, drug–drug interactions, and long-term safety remains uncertain.

### 5.2 Chiglitazar

Chiglitazar further broadens this therapeutic landscape through its distinctive pan-PPAR profile, exerting concurrent agonistic activity on PPARα, PPARγ, and PPARδ. Such a multi-receptor engagement is conceptually attractive, as it enables a more integrated modulation of metabolic pathways and addresses several interconnected pathophysiological mechanisms underlying MASLD in the context of T2D.

#### 5.2.1 Randomized clinical evidence

The clinical evidence supporting chiglitazar has expanded substantially in recent years. Most notably, a multicenter phase II randomized, double-blind, placebo-controlled trial in patients with MASLD, hypertriglyceridemia, and insulin resistance demonstrated significant reductions in liver fat content assessed by MRI-PDFF after 18 weeks of treatment, together with improvements in liver enzymes and metabolic parameters. The reduction in hepatic fat was dose-dependent, and the drug was generally well tolerated, with predominantly mild-to-moderate adverse events. These findings provide the strongest clinical evidence to date supporting the therapeutic potential of pan-PPAR activation in MASLD (Tan et al., 2025; Sun et al., 2026).

#### 5.2.2 Real-world evidence and subgroup analyses

Prospective real-world data in patients with T2D and MASLD have corroborated these findings, showing greater reductions in hepatic steatosis compared with alternative glucose-lowering therapies. Importantly, treatment effects appeared consistent across age, sex, body mass index, and concomitant use of SGLT2 inhibitors or GLP-1 RAs, suggesting broad applicability in heterogeneous patient populations.

#### 5.2.3 Drug tolerability and safety

Data from randomized clinical trials indicate that chiglitazar is generally well tolerated. In the phase II placebo-controlled trial, treatment-emergent adverse events were predominantly mild to moderate and occurred at rates comparable to placebo. Urinary tract infection, hyperuricemia, and transient infectious illnesses were the most frequently reported events, whereas serious treatment-related adverse events were not observed (Sun et al., 2026).

### 5.3 Saroglitazar

In MASLD, disruption of hepatic lipid homeostasis is a central driver of atherogenic lipoprotein production. Excessive triglyceride synthesis in the liver generates oversized, lipid-enriched VLDL particles that enter the systemic circulation. These large VLDL particles are metabolized slowly, giving rise to small, dense LDL (sdLDL), a highly atherogenic subfraction strongly linked to cardiovascular events in MASLD patients. Activation of PPARα by saroglitazar mitigates this atherogenic burden by enhancing hepatic fatty acid oxidation, limiting the formation of oversized VLDL, and thereby reducing the substrate for sdLDL generation. Concurrent suppression of apolipoprotein C-III further decreases production of atherogenic lipoproteins (Syed and Siddiqui, 2021; Siddiqui et al., 2015; Patel et al., 2021; Patel and Siddiqui, 2019). While previous studies with pan-PPAR agonists, such as lanifibranor, demonstrated modest improvements in conventional lipid parameters, saroglitazar provides a more nuanced modulation of the atherogenic lipoprotein profile. Its potent PPARα activity, coupled with superior PPARγ engagement relative to pioglitazone or lanifibranor, likely underpins its distinctive lipoprotein effects. Additionally, saroglitazar induces the transcription of multiple PPARγ-responsive genes involved in lipid and glucose metabolism, including adiponectin, adipocyte fatty acid-binding protein, LPL-C, fatty acid transport protein, and fatty acid translocase (CD36). This coordinated transcriptional program attenuates postprandial plasma free fatty acid surges, enhances insulin-mediated suppression of hepatic glucose output, reduces metabolic stress on liver and skeletal muscle, and promotes peripheral glucose utilization (Jain et al., 2018; Kaul et al., 2019; Francque et al., 2021; Boubia et al., 2018; Jain et al., 2015). Although the clinical consequences of these changes require further investigation, LDL-C remains a well-established driver of atherosclerotic risk, and its reduction is a cornerstone of cardiovascular therapeutics. Importantly, sdLDL has emerged as a superior predictor of atherosclerotic burden compared with total LDL-C, and conventional statin therapy may not fully normalize sdLDL despite achieving target LDL-C levels. This is particularly relevant in MASLD, where sdLDL is markedly elevated relative to matched controls.

#### 5.3.1 Clinical evidence

Clinical studies suggest improvements in biochemical markers of liver injury and cardiometabolic risk, supporting its potential role in patients with MASLD associated with dyslipidemia and T2D.

#### 5.3.2 Ongoing clinical development

Available evidence suggests that saroglitazar may possess a comparatively favorable safety profile among currently investigated dual PPAR agonists (Mora et al., 2007; Cali et al., 2007).

#### 5.3.3 Drug tolerability and safety

Across clinical studies and post-marketing analyses, saroglitazar has generally demonstrated a favorable safety profile. Most adverse events were mild and included dyspepsia, gastritis, fatigue, and transient biochemical abnormalities. Importantly, rates of clinically significant edema, heart failure, and excessive weight gain appeared lower than those historically reported with several earlier dual PPAR agonists (Kaul et al., 2019).

#### 5.3.4 Summary and future directions

Among dual PPAR agonists, saroglitazar appears particularly attractive because it simultaneously targets hepatic steatosis and atherogenic dyslipidemia. However, definitive evidence from large, randomized studies with biopsy-based or long-term clinical endpoints is still needed.

### 5.4 LT175

Another dual PPARα/γ agonist is LT175, that has a potent insulin-sensitizing properties. It orchestrates a selective modulation of gene networks governing lipid uptake and storage in adipocytes, thereby mitigating the propensity for weight gain. Concurrently, hepatic PPAR activation by LT175 enhances triglyceride and fatty acid catabolism, promoting efficient lipid clearance and conferring additional metabolic benefits. Collectively, these attributes position LT175 as a highly attractive scaffold for the rational design of next-generation PPAR ligands with optimized metabolic profiles, capable of improving both glycemic control and lipid homeostasis while potentially attenuating cardiovascular risk in individuals with T2D and metabolic syndrome (Gilardi et al., 2014).

#### 5.4.1 Preclinical evidence

Evidence for LT175 remains confined to experimental models. Available studies indicate that this dual PPARα/γ agonist improves insulin sensitivity while selectively modulating adipocyte gene expression and enhancing hepatic fatty acid oxidation, potentially minimizing excessive adipose tissue expansion.

#### 5.4.2 Drug tolerability and safety

Safety information for LT175 is currently restricted to preclinical investigations. Experimental studies have not identified major toxicity signals, but the absence of human clinical data precludes meaningful conclusions regarding tolerability, adverse events, or long-term safety.

### 5.5 Tesaglitazar

#### 5.5.1 Clinical and translational evidence

Tesaglitazar demonstrated metabolic efficacy in early clinical development, but its progress was ultimately limited by safety concerns. More recent translational investigations have focused on targeted drug-delivery approaches, including liposomal formulations, revealing that selective macrophage targeting alone does not reproduce the systemic metabolic benefits achieved with conventional administration (Allen and Cullis, 2013; Sercombe et al., 2015; Song et al., 2014).

#### 5.5.2 Drug tolerability and safety

Despite demonstrating metabolic efficacy in early clinical studies, tesaglitazar development was discontinued because of safety concerns. Clinical trials reported increases in serum creatinine, reductions in estimated glomerular filtration rate, fluid retention, peripheral edema, and weight gain, raising concerns regarding renal and cardiovascular safety. Subsequent experimental strategies using targeted nanoparticle delivery aimed to reduce systemic exposure but have not yet overcome these limitations (Fagerberg et al., 2005).

### 5.6 Aleglitazar

Another dual PPARα/γ agonist is Aleglitazar a synthetic dual agonist of peroxisome proliferator–activated receptor, rationally designed to achieve balanced activation of both nuclear receptors. This dual affinity arises from subtle variations in chemical structure that exploit the highly conserved yet geometrically distinct ligand-binding domains of the PPAR isoforms. The overarching objective of balanced PPARα/γ agonists is to induce more potent PPARα activation than that achieved with fibrates, while maintaining—or enhancing—the insulin-sensitizing and cardiometabolic benefits associated with PPARγ activation, without incurring the adverse effects typically observed with thiazolidinediones.

#### 5.6.1 Randomized clinical trials

Aleglitazar was designed as a balanced dual PPARα/γ agonist and demonstrated favorable effects on glycemic control and lipid metabolism in randomized studies involving patients with T2D. Preliminary clinical evidence suggests that aleglitazar favorably modulates multiple cardiometabolic risk parameters, including glycemic control, high-density and low-density lipoprotein cholesterol, triglycerides, apolipoprotein B levels, and blood pressure. Nonetheless, adverse effects associated with both PPARα and PPARγ activation have been reported.

However, despite these metabolic improvements, subsequent large-scale cardiovascular outcome trials failed to establish an overall favorable risk-benefit profile.

#### 5.6.2 Drug tolerability and safety

The safety profile of aleglitazar has been extensively characterized in randomized clinical trials, including large cardiovascular outcome studies. Although improvements in glycemic control and lipid parameters were consistently observed, treatment was associated with increased rates of heart failure, fluid retention, peripheral edema, weight gain, bone fractures, gastrointestinal events, and renal dysfunction. An excess of adverse events contributed to premature termination of pivotal outcome trials and ultimately halted further clinical development (Lincoff et al., 2014).

## 6 Conclusions

The management of MASLD represents a major challenge in modern medicine due to its multisystemic nature and growing global impact, marking a significant shift from single-receptor activation to the use of dual and triple agonists as a comprehensive multi-target strategy. The data analyzed in this manuscript demonstrate that co-agonists such as GLP-1/GIP and GLP-1/GCG, as well as tri-agonists like GLP-1/GIP/GCG, synergistically enhance weight reduction and directly address liver damage by mitigating IR, oxidative stress, and inflammation (Table 1). While molecules like tirzepatide and retatrutide have shown an unprecedented ability to reduce intrahepatic lipid content and potentially reverse fibrosis, several challenges remain for the future, including the need for long-term efficacy data to ensure that histological improvements translate into a reduction in major clinical events such as cirrhosis and hepatocarcinoma. Furthermore, future research must prioritize the identification of specific biomarkers to enable personalized medicine based on individual metabolic phenotypes, while simultaneously addressing the socio-economic challenges of drug accessibility and reimbursement. Exploring the synergy of these agents with other therapeutic classes, such as thyroid hormone receptor-beta agonists or FGF21 inhibitors, could further refine treatment protocols. Ultimately, dual and triple agonists are not merely weight-loss tools but a transformative frontier in MASLD therapy that holds the potential to radically alter the natural history of the disease, reducing the need for liver transplantation and significantly improving patient survival and quality of life.

**TABLE 1:** Compendium of dual and triple agonism clinical trials.

Type of Agonism	Receptor targets	Molecules	Clinical Trials					
			Name	Study design	Duration	Participants (no)	Comparator	Results
Dual agonism	GLP-1R/GIPR	Tirzepatide	SYNERGY-NASH	Randomized multicenter, double-blind trial	52 weeks	190	Placebo	Tirzepatide significantly improved resolution of MASH without worsening of fibrosis
	​	​	NCT06934642	Interventional	N/A	N/A	N/A	Identification of an changes in biomarkers associated with MASLD with tirzepatide use
	​	​	SYNERGY-outcomes	Interventional	N/A	4,500 approx	Placebo, retatrutide	Retatrutide and tirzepatide can prevent major adverse liver outcomes (MALO) in people with MASLD
	GLP-1R/GCGR	Cotadutide	NCT03235050	Randomized, double-blind, phase 2b trial	54 weeks	834	Placebo, liraglutide	Cotadutide demonstrated modest but statistically significant reductions in body weight and improvements in glycaemic control compared with placebo
		Mazdutide	NCT04904913	Randomized, two-part (low doses up to 6 mg and high dose of 9 mg), double-blind, phase 2 trial	24 weeks	248	Placebo	Mazdutide is associated with weight loss, improvements in glycaemic control, and favourable changes in cardiovascular risk factors (including blood pressure and lipid profile)
		Mazdutide	Not available	Randomized, double-blind, phase 2 trial	20 weeks	252	Placebo, dulaglutide	Mazdutide is associated with weight loss, improvements in glycaemic control, and favourable changes in cardiovascular risk factors (including blood pressure and lipid profile)
		Pemvidutide	NCT05006885	Randomized, double-blind, clinical trial	12 weeks	94	Placebo	Pemvidutide is associated with reduction of liver fat content, serum ALT and body weight
		Pemvidutide	Not available	Randomized, double-blind, clinical trial	24 weeks	64	Placebo	Pemvidutide is associated with reduction of liver fat content, serum ALT and body weight
		Pemvidutide	IMPACT	Randomised, double-blind, phase 2b trial	48 weeks	212	Placebo	Pemvutide is associated with reductions in liver fat content and improvements in selected hepatic parameters, without clinically meaningful hepatotoxicity or deterioration in glycaemic control
		Survodutide	NCT04667377	Randomised, double-blind, dose-finding phase 2 trial	46 weeks	386	Placebo	Survodutide is associated with significant weight loss, with additional favourable changes in selected cardiometabolic parameters such as waist circumference and blood pressure
		Survodutide	NCT04153929	Randomised, double-blind, parallel-group, phase 2 trial	16 weeks	413	Placebo, semaglutide	Survodutide is associated with dose-dependent reductions in HbA1c and body weight, superior to placebo and comparable to semaglutide, with a GLP-1–like safety profile
		Survodutide	NCT05296733	Non-randomized, open-label, phase I clinical trial	31 weeks	40	None	Survodutide demonstrated acceptable tolerability and pharmacokinetics in compensated cirrhosis, with no clinically relevant safety concerns
	Dual amylin–calcitonin receptors	Cagrilintide	NCT03856047	Randomized, double-blind, phase 2 trial	26 weeks	706	Placebo, liraglutide	Overweight and obesity people had a significant reductions in bodyweight
	​	​	REDEFINE 1	Randomized, double-blind, placebo-controlled, parallel-group	68 weeks	3,417	Placebo, Semaglutide	Cagrilintide–semaglutide provided significant and clinically relevant body-weight reductions in adults with overweight or obesity
	​	​	REDEFINE 2	Randomized, double-blind, placebo-controlled	68 weeks	1,206	Placebo	Once-weekly cagrilintide– semaglutide (at a dose of 2.4 mg each) resulted in a significantly lower body weight than placebo in adults with obesity and type 2 diabetes
	PPAR-α/γ agonist	Saroglitazar	NCT03061721	Randomized, double-blind, phase 2 trial	16 weeks	106	Placebo	Reduction of ALT and liver fat on MRI
		Tesaglitazar	GALLANT 9	Randomized, double-blind, phase 3 trial	24 weeks	370	Placebo	In addition to insulin, reduction in HbA1c Interrupted
		Aleglitazar	ALECARDIO	Randomized, double-blind, phase 3 trial	104 weeks	7,226	Placebo	No significant reduction in cardiovascular risk. Improvements in HbA1c, HDL, and triglycerides occurred, but with weight gain, edema, renal events, and other adverse effects Interrupted
			ALENEPHRO	Randomized, double-blind, phase IIb trial	52 weeks	150	Pioglitazone	Non-inferiority of change in eGFR
Triple agonism	GLP-1R/GIPR/GCGR	Retatrutide	NCT04867785	Phase 2, double-blind, randomized controlled trial	Over 36 weeks	281	Placebo Dulaglutide	Greater reductions in body weight, adiposity, perceived appetite, hunger, and tendency to overeat, HbA1c, compared to placebo and active comparator
		​	NCT05567796	Secondary analysis of a phase 2 randomized controlled trial	Over 48 weeks	338	Placebo	Superior to placebo in weight loss
		​	TRIUMPH	Phase 3 randomized, double-blind, trial	NA	NA	Placebo	Ongoing
		Efocipegtrutide	HM-TRIA-201	Phase 2 adaptive trial	NA	NA	Placebo	Ongoing
	Pan-PPAR agonist	Chiglitazar (Rubusoside)	CMAP	Randomized, double-blind, phase 3 trial	24 weeks	535	Placebo	Primary end point: reduction of HbA1c Secondary end points: reduction of glycemia and triglycerides and increased insulin sensitivity
			CMAS	Randomized, double-blind, phase 3 trial	24 weeks	739	Sitagliptin	Non-inferior to sitagliptin in reducing HbA1c
			RECAM	Randomized, double-blind, phase 3 trial	24 weeks	533	Placebo	In addition to metformin, reduction in HbA1c
